# Neurological complications in COVID-19 – a new diagnostic challenge

**DOI:** 10.25122/jml-2022-0056

**Published:** 2022-02

**Authors:** Vitalie Vacaras, Sorina Frunze, Mihai Adrian Cordos

**Affiliations:** 1. Department of Neurosciences, Iuliu Hatieganu University of Medicine and Pharmacy, Cluj-Napoca, Romania; 2. Neurology Department, County Emergency Hospital, Cluj-Napoca, Romania

To the Editor,

After reading the letter sent by Finsterer J *et al.* regarding our previously published article, “Neurological complications in COVID-19 – a diagnostic challenge”, we decided to offer some explanations. First of all, we would like to remind readers that this article was published in April 2021, and the patient whose case we presented was hospitalized about 6 months prior to publication. At that time, the COVID – 19 pandemic was in its early stages, and knowledge of both the pathophysiological mechanisms involved in this disease and therapeutic resources was limited. In the following, we explain punctually each observation mentioned in the article by Finsterer J *et al.*

• Although digital subtraction angiography (DSA) is the gold-standard method in vascular imaging, being particularly useful for the evaluation of subarachnoid hemorrhage (SAH) without an identified bleeding source on computed tomography (CT-scan) or magnetic resonance imaging (MRI), it is an invasive procedure that carries a risk, albeit minimal, of transient as well as persistent neurological complications [1–3]. In our case, technical possibilities did not allow us to apply this method for a complete evaluation of the cause of SAH. However, some studies published in the literature have shown only minimal advantages of DSA over computed tomography angiography (CTA) [1, 4] in the assessment of SAH and attempt to demonstrate the relative futility of using DSA for SAH [4]. In our case, the head CTA examination was subsequently supplemented with head and neck CTA, thus increasing the probability of detecting certain aneurysms not seen on initial examination.
• The absence of xanthochromia in cerebrospinal fluid (CSF) examination can be explained by the fact that the lumbar puncture was performed 12 days after the onset of bleeding, and the SAH was minimal from the beginning.
• We considered identifying SARS-CoV-2 in the CSF as indispensable to support our hypothesis that the viral infection was the cause of the brain lesions, but we also specified, in our article, the technical limitations at that moment in time. Evaluating the levels of IL-6, IL-8, IL-1beta, TNF-alpha in the CSF was not technically possible either.
• The cardio-embolic etiology of cerebral ischemia could not be totally excluded during hospitalization, but we mentioned in the initial presentation that the patient was referred for a complete cardiological evaluation, including Holter – ECG and cardiac ultrasound, immediately after discharge.
• As for the treatment of SARS-CoV-2 infection, it was not administered during the patient’s stay in our hospital. However, the national guidelines valid at that time of the pandemic recommended using both hydroxychloroquine and ritonavir/lopinavir for the treatment of COVID-19 [5].
• Coagulation was assessed by determining aPTT, Quick time, INR, and PT. These were within the normal range, and for this reason, we did not mention these in the initial presentation.
• The brain MRI examination was performed during hospitalization. As specified in the article, it was performed on the 8^th^ day after the neurological manifestations appeared; the patient was admitted to our unit 5 days after developing these manifestations. The patient was hospitalized for 7 days in our hospital.

We are grateful for the interest expressed in our publication and hope that the above arguments will clarify the omissions previously made. We consider the issues raised useful both for our further practice and for scientific progress.

## Acknowledgements

### Conflict of interest

The authors declare no conflict of interest.
